# Study of Diffusion Weighted Imaging Derived Diffusion Parameters as Biomarkers for the Microenvironment in Gliomas

**DOI:** 10.3389/fonc.2021.672265

**Published:** 2021-10-12

**Authors:** Yan Bai, Taiyuan Liu, Lijuan Chen, Haiyan Gao, Wei Wei, Ge Zhang, Lifu Wang, Lingfei Kong, Siyun Liu, Huan Liu, Neil Roberts, Meiyun Wang

**Affiliations:** ^1^ Department of Medical Imaging, Henan Provincial People’s Hospital and The People’s Hospital of Zhengzhou University, Zhengzhou, China; ^2^ Department of Pathology, Henan Provincial People’s Hospital and The People’s Hospital of Zhengzhou University, Zhengzhou, China; ^3^ Pharmaceutical Diagnostics, General Electric (GE) Healthcare, Beijing, China; ^4^ The Queen’s Medical Research Institute, University of Edinburgh, Edinburgh, United Kingdom

**Keywords:** diffusion weighted imaging, intravoxel incoherent motion, diffusion kurtosis imaging, gliomas, biomarker

## Abstract

**Objectives:**

To explore the efficacy of diffusion weighted imaging (DWI)-derived metrics under different models as surrogate indicators for molecular biomarkers and tumor microenvironment in gliomas.

**Methods:**

A retrospective study was performed for 41 patients with gliomas. The standard apparent diffusion coefficient (ADC_st_) and ADC under ultra-high *b* values (ADC_uh_) (*b* values: 2500 to 5000 s/mm^2^) were calculated based on monoexponential model. The fraction of fast diffusion (*f*), pseudo ADC (ADC_fast_) and true ADC (ADC_slow_) were calculated by bi-exponential model (*b* values: 0 to 2000 s/mm^2^). The apparent diffusional kurtosis (K_app_) was derived from the simplified diffusion kurtosis imaging (DKI) model (*b* values: 200 to 3000 s/mm^2^). Potential correlations between DWI parameters and immunohistological indices (i.e. Aquaporin (AQP)1, AQP4, AQP9 and Ki-67) were investigated and DWI parameters were compared between high- and low-grade gliomas, and between tumor center and peritumor. Receiver operator characteristic (ROC) curve and area under the curve (AUC) were calculated to determine the performance of independent or combined DWI parameters in grading gliomas.

**Results:**

The ADC_slow_ and ADC_uh_ at tumor center showed a stronger correlation with Ki-67 than other DWI metrics. The ADC_st_, ADC_slow_ and ADC_uh_ at tumor center presented correlations with AQP1 and AQP4 while AQP9 did not correlate with any DWI metric. K_app_ showed a correlation with Ki-67 while no significant correlation with AQPs. ADC_st_ (*p* < 0.001) and ADC_slow_ (*p* = 0.001) were significantly lower while the ADC_uh_ (*p* = 0.006) and K_app_ (*p* = 0.005) were significantly higher in the high-grade than in the low-grade gliomas. ADC_st_, *f*, ADC_fast_, ADC_slow_, ADC_uh_, K_app_ at the tumor center had significant differences with those in peritumor when the gliomas grade became high (*p* < 0.05). Involving ADC_uh_ and K_app_ simultaneously into an independent ADC_st_ model (AUC = 0.833) could further improve the grading performance (ADC_st_+ADC_uh_+K_app_: AUC = 0.923).

**Conclusion:**

Different DWI metrics fitted within different *b*-value ranges (low to ultra-high *b* values) have different efficacies as a surrogate indicator for molecular expression or microstructural complexity in gliomas. Further studies are needed to better explain the biological meanings of these DWI parameters in gliomas.

## Introduction

Gliomas are the most common primary brain tumors in adults and according to World Health Organization (WHO) guidelines are classified into four grades, which reflect increasing malignancy and worse prognosis (1). Assays of histopathology and molecular pathology based on tumor samples obtained by resection or biopsy are the gold standard for determining the pathological grade and molecular subtype. Accurate assessment of glioma grade, as well as phenotype and genotype, is of potential importance for the optimization of personalized treatment. However, inherently high heterogeneity of gliomas means that biopsy or localized resection may not be representative of the tumor as a whole.

Improving the non-invasive characterization of glioma physiology or pathology might help to improve the image-guided biopsy and therapy. Considering different regimes of *b*-value could control the degree of diffusion-weighting in the diffusion weighted imaging (DWI), different tissue properties reflected by the water diffusion could be encoded into DWI signals. Therefore, DWI is one of the potential tools to provide surrogate noninvasive imaging biomarkers for microenvironment in gliomas as the water diffusion coefficients could intermediately reflect the microstructure, perfusion or water exchange effects associated with transmembrane transport, such as facilitated diffusion (2, 3).

As the cell proliferation and the water transportation are highly suspected to influence the water diffusion properties in gliomas at extracellular, intracellular or transcellular space, Ki-67 or aquaporin (AQP) subtypes (AQP1, AQP4, AQP9) were welcomed molecular targets quantified by different DWI diffusion metrics (4, 5). Ki-67 is an immunohistochemical marker for the proliferation in gliomas which is known to correlate with tumor grading (4) and prognosis (6). AQPs provide a major pathway for the water transportation through cell membrane (7). And AQP subtypes, including AQP1, AQP4 and AQP9, are overexpressed in glioma cells and correlated with tumor grade and malignancy (8–12). AQP1, AQP4 and AQP9 were reported to be related to angiogenesis, invasion and peritumoral edema in gliomas. AQP1 predominantly locates in the perivascular space and it has been reported that increased AQP1 might induce vasogenic brain edema (13) and acceleration of cell migration and invasion (10, 14). AQP4 are mainly expressed by astrocytes and its redistribution is thought to control water mobility at the blood-brain interface and progress along with blood-brain barrier disturbance and vascular proliferation (11, 15, 16). Increased expression of AQP9 has been observed in glioma tissue near vessels and might promote the invasion and motility of cells (17, 18).

Several studies have demonstrated that some diffusion coefficients derived from intravoxel incoherent motion (IVIM), diffusion kurtosis imaging (DKI) or stretched-exponential models such as slow apparent diffusion coefficient (ADC_slow_), axial kurtosis and heterogeneity index α could correlate with Ki-67 expression in gliomas (4, 19, 20), whilst the DKI-derived mean kurtosis, diffusion tensor imaging-derived mean diffusivity, the apparent ADC and ADC derived from ultra-high *b*-value model showed different correlation tendency with AQP in gliomas (5, 21). However, the levels of the correlations seemed to be diverse and few studies have compared the efficacy of different kinds of model-derived parameters for indicating Ki-67 and AQP in gliomas among the same dataset.

Considering more DWI models to select more rational or efficient diffusion surrogate biomarkers for indicating molecular expression or phenomenologically depicting the gliomas microenvironment is necessary at present. In the current study, the diffusion metrics derived from mono-exponential model, IVIM model, DKI model and ultra-high *b*-value model, and their efficacy for quantifying Ki-67, AQP1, AQP4, and AQP9 were analyzed and compared. Among these models, the mono-exponential model could reflect apparent diffusion combining diffusion and perfusion; IVIM model could separate the slow diffusion in response to intra- or extracellular water molecules from the fast diffusion in response to tubular or vascular perfusion; DKI model could help provide general structure information and quantitative information about diffusion deviation from freely gaussian diffusion; and ultra-high *b*-value model could provide the diffusion information in the specific high *b*-value range which is potential to indicate transmembrane diffusion (2, 3). By comprehensively considering these models covering gaussian or non-gaussian assumption and *b*-value compartment, we expected to contribute as many shreds of evidence as possible during the formation of DWI biomarkers for the gliomas microenvironment. In addition, as the AQPs distribution indicates the disease progression (9, 22), the spatial distribution of the diffusion coefficients might noninvasively indicate physiological or pathological conditions related to the change of water exchange or transportation. Therefore, the same set of diffusion metrics from central and peritumoral regions of low- and high-grade gliomas were also analyzed and compared to help depict a more complete tumor microenvironment picture.

## Materials and Methods

### Patients Population

This retrospective study was approved by the local institutional review board, and written informed consent was obtained from each patient. A total of 45 patients with pathologically proven gliomas diagnosed between October 2014 and May 2016 were enrolled in the study. The inclusion criteria were as follows: (a) MRI examinations were performed on patients prior to treatments of tumors and (b) the pathological diagnoses and histological indices were acquired by surgical resections of gliomas. Four patients were excluded due to the presence of head movement artifacts in the DWI images. The final analysis was performed for a total of 41 patients with gliomas.

### MRI Data Acquisition

MRI investigations were performed for all patients by using a Discovery MR 750 3 T MRI system (GE Healthcare, Milwaukee, Wisconsin, USA). Firstly, T1-weighted (T1w) images were acquired using an Inversion-Recovery Fast Spin-Echo sequence with repetition time (TR) of 1593 msec and echo time (TE) of 24 msec, and T2-weighted (T2w) images were obtained by using a FSE sequence (TR/TE, 4600 msec/110 msec). Next, DWI was performed using a single-shot, echo-planar sequence (TR/TE, 4000 msec/112 msec; matrix, 128 × 128; Field of View (FOV), 24 × 24 cm^2^; and slice thickness, 4 mm). A total of 16 *b* values were acquired in three orthogonal directions at 0, 50, 100, 150, 200, 300, 400, 500, 800, 1000, 1500, 2000, 2500, 3000, 4000 and 5000 sec/mm^2^. Total acquisition time for DWI was 5 minutes 32 seconds. Finally, the T1w sequence was repeated after intravenous administration of 0.1 mmol/kg gadopentetate dimeglumine.

### MRI Data Analysis

Two radiologists, blinded to the reports concerning tumor pathology, reviewed and analyzed all the MR images independently on a remote workstation. The radiologists independently drew three different regions of interests (ROIs) on the T2w echo-planar image for each tumor in the solid parts and regions within 1 cm peritumoral parts, respectively. Each MRI parameter in the solid parts or peritumoral region were determined by the averaged value of three ROIs, respectively. Areas of necrosis, hemorrhage and cerebrospinal fluid were excluded to ensure accurate measurements.

### DWI Data Processing

DWI data were transferred to a workstation (Advantage Workstation 4.5; GE Healthcare) for processing.

ADC_st_ was calculated from *b* values of 0 and 1000 sec/mm^2^ by using a monoexponential DWI model (23) as Equation (1):


Eq. (1) $$\text{S}(b)/\text{S}(0)=\exp(-b\times \text{AD}{\text{C}}_{\text{st}}),$$

where S(*b*) represents the signal intensity in the presence of diffusion sensitization, and S(0) represents the signal intensity in the absence of diffusion sensitization. This model used the least square fit for linear fitting (24).

ADC_slow_ was obtained from IVIM model with *b* values from 0 to 2000 sec/mm^2^ (23) as Equation (2):


Eq. (2) $$\text{S}(b)/\text{S}(0)=f\times \exp(-b\times \text{ AD}{\text{C}}_{\text{fast}})+(1-f)\times \exp(-b\times \text{ AD}{\text{C}}_{\text{slow}}),$$

where *f* represents the fraction of fast diffusion component, ADC_fast_ represents the pseudo-diffusion coefficient, and ADC_slow_ represents the slow diffusion coefficient. The Levenberg-Marquardt fit was used for nonlinear fitting (24).

ADC_uh_ under ultra-high *b* values was calculated from *b* values of 2500, 3000, 4000 and 5000 sec/mm^2^ by using the above monoexponential DWI model (25) as Equation (1).

Apparent diffusional kurtosis (K_app_) was calculated from DKI model (26) according to Equation (3) with *b*-value ranging from 200 to 3000 sec/mm^2^:


Eq. (3) $$S(b)/S(0)=\exp(-b\cdot D_{app}+\frac{1}{6}b^{2}\cdot D_{app}^{2}\cdot K_{app}),$$

where D_app_ (unit: ×10^-3^ mm^2^/s) is the apparent diffusion coefficient fitted in low *b*-value range of 200-1000 sec/mm^2^, K_app_ (unitless) is the apparent diffusional kurtosis which is fitted with *b*-value up to 3000 sec/mm^2^.

### Immunohistochemistry

Specimens acquired from surgical resections were embedded in paraffin. AQP1, AQP4, AQP9 and Ki-67 immunohistostainings were conducted for quantification analyses. Slides were rinsed in phosphate buffer saline and blocked with 5% normal goat serum, followed by incubation with primary mouse monoclonal anti-AQP1 antibodies (ab9566, Abcam, Cambridge, UK), mouse monoclonal anti-AQP4 antibodies (ab11026, Abcam, Cambridge, UK), rabbit polyclonal anti-AQP9 antibodies (ab85910, Abcam, Cambridge, UK) or mouse monoclonal mouse anti-Ki-67 (ZM0165, Zhongshan Biotechnology Co., Ltd., Beijing, China) for 2 hours at 37°C. Slides were then incubated with horseradish peroxidase-conjugated secondary antibody diamino-benzidine (Fuzhou Maixin Biotechnology Development Co., Ltd., Fuzhou, China) for 10 minutes at 37°C, and visualized with diaminobenzidine substrate (Fuzhou Maixin Biotechnology Development Co., Ltd., Fuzhou, China).

### Immunohistochemistry Data Analysis

Specimens acquired from surgical resections were embedded in paraffin and immunohistochemistry stains were applied for quantification of AQP1, AQP4, AQP9 and Ki-67. Histological indices of AQP1, AQP4, AQP9 and Ki-67 were independently measured by two pathologists by using a HMIAS-2000 Medical Color Image Analysis System (Champion Image Engineering Co., Ltd., Wuhan, China). The pathologists independently placed three different ROIs in the solid parts of each tumor for each patient. The ROIs excluded the areas of necrosis and hemorrhage. The IODs were measured for AQP1, AQP4, AQP9 and Ki-67, which were averaged from three delineated ROIs.

### Statistical Analysis

MedCalc software (version 19.0, MedCalc, Belgium) and R software (version 3.5.1) were used for statistical analyses. Correlations between DWI-derived parameters and histological indices were computed by using the Pearson’s correlation analysis. Steiger’s Z-test was used for comparing each two correlation coefficients (27). A correlation coefficient (*r*) of 0.75–1.00 was set to indicate very good to excellent correlation; 0.50–0.74, moderate to good correlation; 0.25–0.49, fair correlation; and 0.24 or lower indicate little or no correlation (28, 29). The Mann-Whitney *U* test was used for statistically comparing parameters between high-grade and low-grade gliomas. The significant difference of DWI-derived parameters between tumor center and peritumoral regions was analyzed by using the intragroup paired *t*-test or Wilcoxon matched-pairs signed rank test. A receiver operator characteristic (ROC) curve and area under the ROC curve (AUC) were calculated to evaluate the model performance in grading gliomas. The maximum Youden index was used to determine the threshold for calculating the sensitivity and specificity. AUC of the paired models were compared by Delong’s test. The continuous net reclassification improvement (NRI), and integrated discrimination improvement (IDI) indices were analyzed to assess the added value of combined models (30). Interobserver agreement for each measurement was calculated by using an Intraclass Correlation Coefficient (ICC) with 95% confidence interval (CI). Two sides *p* values less than 0.05 were considered statistically significant. The R packages were mainly involved as follows: “icc” was used for ICC calculation by setting “twoway” and type of “agreement”, “glmnet” package for logistic regression, “pROC” package for ROC analysis, “PredictABEL” was used for NRI and IDI evaluation, and “ggboxplot” for boxplot.

## Results

### Patients

26 of 41 included patients (63.4%) were confirmed to have high-grade gliomas (grade 3 and grade 4) and the remaining 15 patients (36.6%) were confirmed to have low-grade (grade 2) gliomas.

### Analysis of Interobserver Agreement Analysis

The ICC between the two independent pathologists for measuring the IODs of AQP1, AQP4, AQP9 and Ki-67 were 0.861 (95% CI = 0.740-0.926), 0.853 (95% CI = 0.724-0.922), 0.841 (95% CI = 0.702-0.915) and 0.877 (95% CI = 0.769-0.934), respectively. The ICC between the two independent radiologists’ calculation of ADC_st_, f, ADC_fast_, ADC_slow_, ADC_uh_, K_app_ values in the tumor center and peritumoral region were summarized in **Table S1**. All of the derived DWI metrics showed good to excellent inter-observer agreement (ICC > 0.8).

### Differences in DWI-Derived Parameters and Histological Indices Between High-Grade and Low-Grade Gliomas

**Figure 1:**
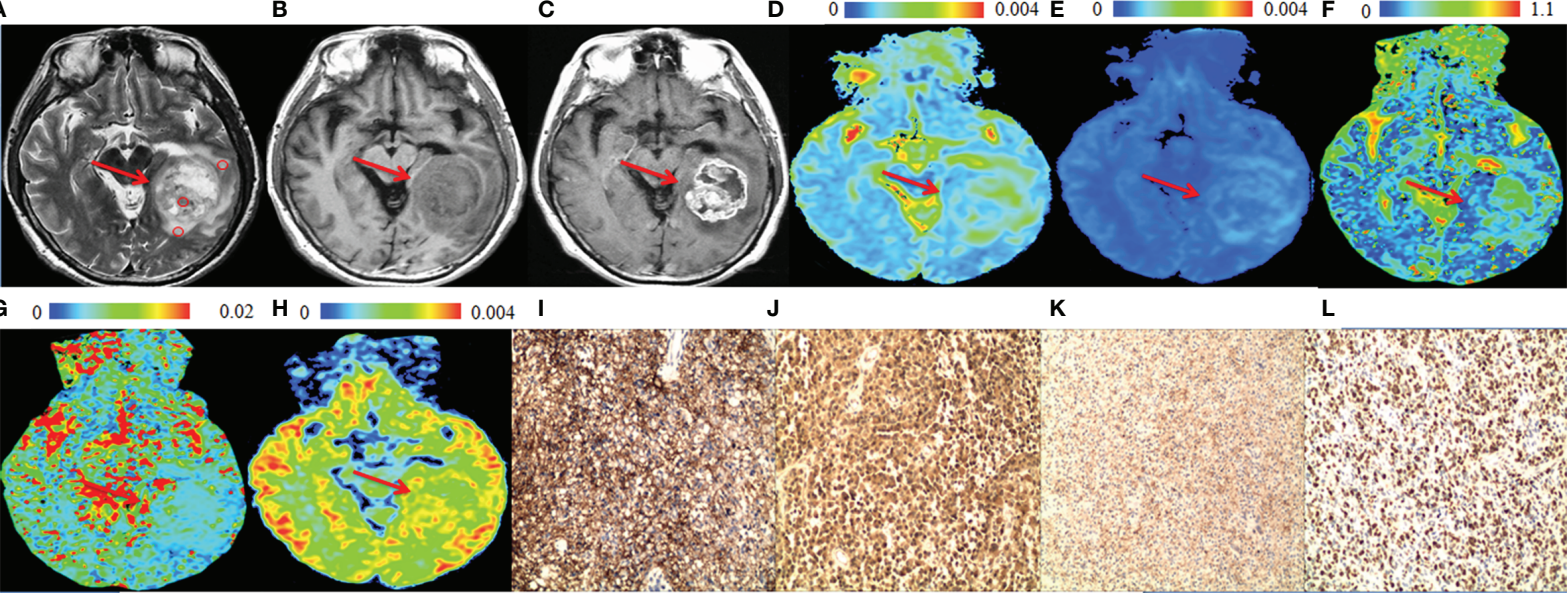
A 57-year-old male patient with a representative glioblastoma (World Health Organization grade 4). In the solid part of the tumor (arrows), **(A)** T2-weighted image shows isointense to hyperintense (circles are the regions of interest), **(B)** T1-weighted image shows hypointense. **(C)** Post-gadolinium T1-weighted image shows enhancement of the solid part of the tumor. **(D)** apparent diffusion coefficient (ADC_slow_) map and **(E)** slow diffusion coefficient (ADC_slow_) map show no increase of the values in the solid part of the tumor. **(F)** fraction of fast diffusion component **(F)** map show no increase of the values in the solid part of the tumor. **(G)** pseudo-diffusion coefficient (ADC_fast_) map shows increased values in the solid part of the tumor. **(H)** ADC with ultra-high *b* values (ADC_uh_) map shows increased value in the solid part of the tumor. **(I)** aquaporin (AQP)1, **(J)** AQP4 and **(K)** Ki-67 immunohistostaining maps show increased expression, whereas **(L)** AQP9 immunohistostaining map shows no obvious increased expression (original magnification, ×100).

**Figure 2:**
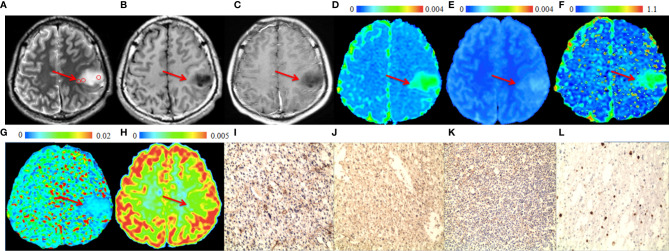
A 28-year-old male patient with a representative astrocytoma (World Health Organization grade 2). In the tumor (arrows), **(A)** T2-weighted image shows hyperintense (circles are the regions of interest), **(B)** T1-weighted image shows hypointense. **(C)** Post-gadolinium T1-weighted image shows no enhancement of the tumor. **(D)** ADC map and **(E)** ADC_slow_ map show increased values in the tumor. **(F)** f map shows increased values in the tumor. **(G)** ADC_fast_ map show no increase in the values in the tumor. **(H)** ADC_uh_ map shows no obvious increase in the value in the tumor. **(I)** AQP1, **(J)** AQP4, **(K)** Ki-67 and **(L)** AQP9 immunohistostaining maps show no obvious increase in expression (original magnification, ×100).

The significant differences in DWI-derived imaging parameters and histological indices between the high-grade and low-grade gliomas are listed in **Table 1**. Values of ADC_st_ and ADC_slow_ at the center of the tumor were significantly lower in high-grade gliomas than in low-grade gliomas, whereas the value of ADC_uh_ at the center of the tumor was significantly increased in the high-grade gliomas than in the low-grade gliomas. Significant higher K_app_ at tumor center were found in high-grade gliomas than in low-grade gliomas. Analysis of the histological indices showed that the expression of AQP1, AQP4 and Ki67 were significantly higher in the high-grade than in the low-grade gliomas, whereas the expression of AQP9 showed no significant difference between the high-grade and low-grade gliomas. The MRI and immunohistochemistry staining of the high-grade and low-grade gliomas are illustrated in **Figures 1**, **2**, respectively.

**Table 1:** The statistical difference analysis of DWI-derived metrics and histological indices between low- and high-grade gliomas.

Features	Low-grade (min, max)	High-grade (min, max)	Statistics	P-value
ADC_st__center^a^	1.31 (1.16, 1.77)^c^	0.92 (0.85, 1.08)	3.519	*<0.001
ADC_st__peri^b^	1.58 (0.89, 2.09)	1.50 (1.22, 1.73)	0.257	0.797
*f*_center	0.28 (0.17, 0.41)	0.23 (0.17, 0.28)	1.218	0.223
*f*_peri	0.31 (0.21, 0.52)	0.33 (0.22, 0.36)	0.257	0.797
ADC_fast__center	0.40 (0.34, 0.45)	0.45 (0.38, 0.52)	-0.92	0.357
ADC_fast__peri	0.36 (0.35, 0.39)	0.33 (0.31, 0.38)	1.665	0.096
ADC_slow__center	1.13 (0.93, 1.37)	0.81 (0.66, 0.98)	3.343	*0.001
ADC_slow__peri	1.18 (0.71,1.51)^d^	1.13 (0.93,1.42)	0.425	0.675
ADC_uh__center	0.24 (0.20, 0.32)	0.32 (0.28, 0.34)	-2.747	*0.006
ADC_uh__peri	0.11 (0.10, 0.12)	0.11 (0.10, 0.12)	0.514	0.607
K_app__center	0.47 (0.41, 0.50)	0.53 (0.50, 0.62)	-2.801	*0.005
K_app__peri	0.41 (0.36, 0.46)	0.41 (0.38, 0.46)	-0.392	0.695
AQP1	0.14 (0.08,0.21)	0.26 (0.18,0.32)	-5.315	*<0.001
AQP4	0.17 (0.11,0.25)	0.24 (0.17,0.31)	-3.263	*0.002
AQP9	0.11 (0.08, 0.15)	0.07 (0.04, 0.13)	1.57	0.116
Ki-67	0.20 (0.11,0.31)	0.46 (0.32,0.65)	-5.918	*<0.001

ADC, apparent diffusion coefficient; ADC_st_, standard apparent diffusion coefficient; ADC_uh_, apparent diffusion coefficient under ultra-high b values; ADC_fast_, pseudo-diffusion coefficient; ADC_slow_, slow diffusion coefficient; f, the fraction of fast diffusion component; Dapp, apparent diffusion coefficient in the unit of um^2^/s; Kapp, apparent diffusional kurtosis.

a parameter measured from tumor center.

b parameter measured from regions of 1 cm peritumoral parts of the tumors.

c The variables with abnormal distribution were depicted by median (interquartile range, IQR).

d The variables with abnormal distribution were depicted by mean ± SD.

*P-value < 0.05 indicated statistical significance.

### Correlation Between DWI-Derived Parameters and Histological Biomarkers

**Figure 3:**
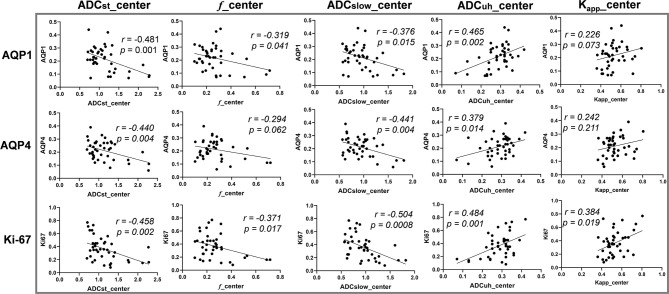
The correlation between diffusion weighted imaging (DWI) parameters and histological indices. Vertical coordinates in rows from top to bottom: AQP1, AQP4 and Ki-67 accordingly. Horizontal coordinates in columns from left to right: ADC_st__center, f_center, ADC_slow__center, ADC_uh__center and K_app__center accordingly. Center: parameter measured from tumor center.

The correlation analysis between DWI-derived imaging parameters and histological indices revealed that expression of AQP1, AQP4 and Ki67 were correlated with ADC_st_, *f*, ADC_slow_ and ADC_uh_ at the center of the tumor while had little correlation with those of peritumoral imaging parameters (see **Table 2** and **Figure 3**). As shown in **Table 2**, the ADC_st_ at the center of the tumor showed a small negative correlation with AQP1, AQP4 and Ki67 (*p* < 0.05). The *f* coefficient at the center of the tumor showed a small negative correlation with AQP1 and Ki67 (*p* < 0.05). The ADC_slow_ at the center of the tumor showed a small negative correlation with AQP1, AQP4 and a moderate negative correlation with Ki-67 (*p* < 0.05). The ADC_uh_ at the center of the tumor showed a small positive correlation with AQP1, AQP4 and Ki67. The DWI metric of Kapp at the center of the tumor showed weak positive and moderate negative correlation with Ki-67 expression (*p* < 0.05), while its correlation with AQPs was not obvious. There was no significant correlation between the expression of AQP9 and any of the imaging parameters (*p* > 0.05).

**Table 2:** Results of correlation analysis between DWI-Derived imaging metrics and histological indices of AQP1, AQP4, AQP9 and Ki-67 molecular expression.

*Correlation*	*AQP1*	*P-value (AQP1)^c^*	*AQP4*	*P-value (AQP4)^d^*	*AQP9*	*P-value (AQP9)^e^*	*Ki-67*	*P-value (Ki-67)^f^*
*ADC_st__center ^a^*	-0.481	*0.001	-0.439	*0.004	-0.037	0.818	-0.458	*0.002
*ADC_st__peri^b^*	-0.134	0.400	0.090	0.572	-0.287	0.068	-0.109	0.497
*f_center*	-0.319	*0.041	-0.293	0.062	-0.004	0.977	-0.370	*0.017
*f_peri*	-0.181	0.255	0.127	0.428	-0.245	0.121	-0.113	0.480
*ADC_fast__center*	-0.170	0.287	0.227	0.152	-0.010	0.949	0.111	0.487
*ADC_fast__peri*	-0.124	0.438	-0.132	0.408	0.015	0.924	-0.202	0.204
*ADC_slow__center*	-0.375	*0.015	-0.441	*0.003	0.087	0.586	-0.503	*0.001
*ADC_slow__peri*	-0.156	0.329	0.064	0.688	-0.251	0.113	-0.114	0.477
*ADC_uh__center*	0.464	*0.002	0.379	*0.014	-0.163	0.307	0.484	*0.001
*ADC_uh__peri*	0.079	0.621	0.108	0.499	0.086	0.590	-0.118	0.461
*K_app__center*	0.226	0.073	0.242	0.211	0.046	0.920	0.384	*0.019
*K_app__peri*	-0.079	0.450	-0.181	0.625	0.314	0.832	-0.011	0.764

ADC, apparent diffusion coefficient; ADC_st_, standard apparent diffusion coefficient; ADC_uh_, apparent diffusion coefficient under ultra-high b values; ADC_fast_, pseudo-diffusion coefficient; ADC_slow_, slow diffusion coefficient; f, the fraction of fast diffusion component; K_app_, apparent diffusional kurtosis derived from DKI model.

a parameter measured from tumor center.

b parameter measured from regions of 1 cm peritumoral parts of the tumors.

c P-value calculated from Pearson’s correlation between AQP1 and each DWI-derived parameter.

d P-value calculated from Pearson’s correlation between AQP4 and each DWI-derived parameter.

e P-value calculated from Pearson’s correlation between AQP9 and each DWI-derived parameter.

f P-value calculated from Pearson’s correlation between Ki-67 and each DWI-derived parameter.

*P-value < 0.05 indicated statistical significance.

In addition, the correlation analysis results between each pair of DWI-metrics derived from tumor center were summarized in **Table S2**. The ADC_st_ showed significant correlation (*p* < 0.05) with other DWI metrics (*f*, ADC_slow_, ADC_uh_ and K_app_), while ADC_uh_ had no significant correlation (*p* > 0.05) with ADC_slow_ and K_app_. The Steiger’s Z test results by comparing different DWI metrics’ correlations with the same histological biomarker were also summarized in **Table S3**. It indicated that there was no significant difference between ADC_st_ and ADC_slow_ for its respective correlation with an individual molecule. While significant differences existed between ADC_uh_ and ADC_st_ or ADC_slow_ because of the inverse correlation tendency.

### Differences in Imaging and Histological Biomarkers Between Tumor Center and Peritumoral Areas

**Figure 4:**
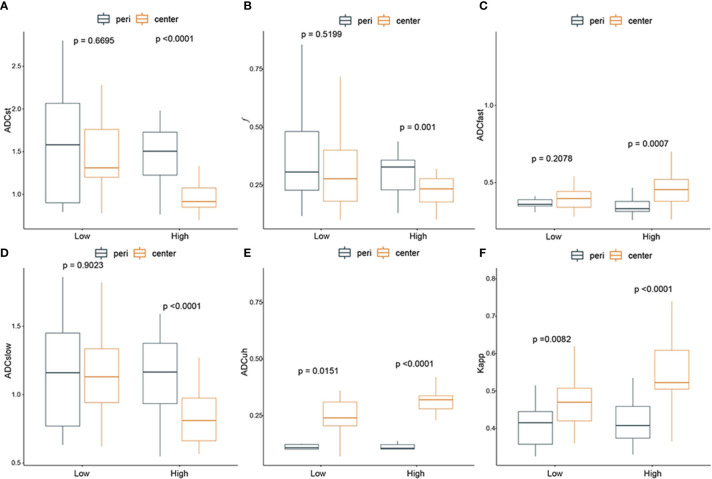
The difference between diffusion weighted imaging (DWI) parameters at the center of the tumor and peritumor in low- and high-grade gliomas. From **(A–F)**: ADC_st_, *f*, ADC_fast_, ADC_slow_, ADC_uh_, K_app_. Black box: peritumoral region; yellow box: tumor center. P-value < 0.05 indicated statistical significance.

The DWI-derived imaging parameters at the center of the tumor and peritumoral area in low- and high-grade gliomas were statistically analyzed as shown in **Figure 4**.

In summary, all the DWI-imaging parameters (including *f*, ADC_st_, ADC_fast_, ADC_slow_, ADC_uh_ and K_app_) at the tumor center had significant differences compared with those in peritumoral areas when the glioma grade became high. And the ADC_uh_ and K_app_ at tumor center were significantly higher than that of peritumor in both low-grade gliomas and high-grade gliomas.

### Diagnostic Performance of Imaging and Histological Biomarkers for Differentiation Between Low- and High-Grade Gliomas

**Figure 5:**
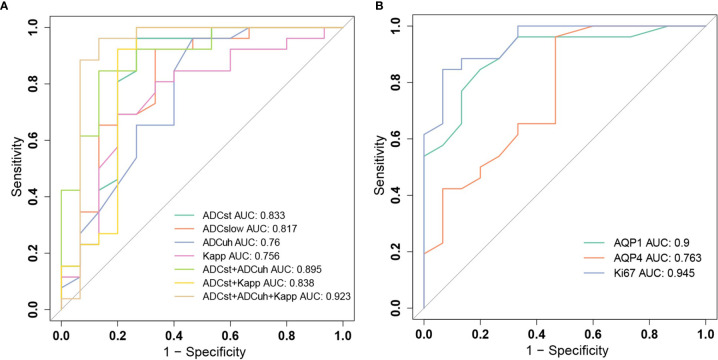
Receiver operating characteristic (ROC) curves for grading gliomas. **(A)** ROC curves of diffusion weighted imaging (DWI) parameters ADC_st_, ADC_slow_, ADC_uh_, K_app_ and their combinations. **(B)** ROC curves of histological biomarkers of AQP1, AQP4 and Ki-67.

The ROC analysis for significant DWI imaging metrics measured from tumor center and histological biomarkers in discriminating high-grade gliomas from low-grade gliomas were conducted and compared as shown in **Figure 5** and **Table 3**.

**Table 3:** The performance of diffusion weighted imaging derived imaging metrics at tumor center and histological biomarkers in differentiating low- and high-grade gliomas.

Parameter	AUC (95%CI)	Optimal cutoff value	Sensitivity (%)	Specificity (%)
ADC_st_	0.833 (0.677-0.990)	1.23^a^	96.15	73.33
ADC_slow_	0.817 (0.667-0.966)	1.03^a^	92.31	66.67
ADC_uh_	0.760 (0.592-0.928)	0.24^a^	96.15	53.33
K_app_	0.756 (0.593-0.920)	0.512	69.2	80.0
ADC_st_ +ADC_slow_	0.851 (0.696-1.0)	-0.026	96.2	73.3
ADC_st_ + ADC_uh_	0.895 (0.793-0.996)	0.702	84.6	86.7
ADC_st_ + K_app_	0.838 (0.672-1.0)	-0.212	100	73.3
ADC_slow_ + ADC_uh_	0.867 (0.734-1.0)	1.064	73.1	93.3
ADC_slow_ + K_app_	0.821 (0.674-0.966)	0.275	88.5	66.7
ADC_uh_ + K_app_	0.882 (0.741-1.0)	0.172	100	80.0
ADC_st_ + ADC_slow_ + ADC_uh_	0.895 (0.765-1.0)	0.007	100	80.0
ADC_st_ + ADC_uh_ + K_app_	0.923 (0.797-1.0)	0.216	96.2	86.7
ADC_st_ + ADC_slow_ + K_app_	0.844 (0.680-1.0)	-0.012	96.2	73.3
ADC_slow_ + ADC_uh_ + K_app_	0.897 (0.767-1.0)	0.704	88.5	86.7
AQP1	0.9 (0.804-0.996)	0.19	84.62	80
AQP4	0.763 (0.603-0.923)	0.14	96.15	53.33
Ki-67	0.945 (0.881-1.0)	0.34	84.62	93.33

AUC, area under curve; ADC, apparent diffusion coefficient; ADC_st_, standard apparent diffusion coefficient; ADC_uh_, ADC under ultra-high b values; ADC_fast_, pseudo-diffusion coefficient; ADC_slow_, slow diffusion coefficient; K_app_, apparent diffusional kurtosis derived from DKI model.

a in units of ×10^-3^ mm^2^/s.

Among molecular biomarkers, Ki-67 presented a good diagnostic ability with the largest AUC and good sensitivity and specificity, which was followed by AQP1. AQP4 presented the best sensitivity whereas poor specificity was determined at a threshold of 0.14. For DWI imaging biomarkers, the ADC_st_ had the largest AUC and relatively better sensitivity and specificity which was followed by ADC_slow_. The ADC_uh_ and K_app_ performed with a similar AUC of around 0.76. While ADC_uh_ had a good sensitivity the same as the ADC_st_ at a threshold of 0.24 and K_app_ had a better specificity (80%) at a threshold of 0.512 compared with other independent imaging metrics. Meanwhile, we assessed the discrimination performance by combining two to three DWI metrics among ADC_st_, ADC_slow_, ADC_uh_ and K_app_. From the aspect of AUC performance, the (ADC_st_ + ADC_uh_: AUC = 0.895) and (ADC_uh_ + K_app_: AUC = 0.882) represented better improvement among two-parameter-based combination models, and (ADC_st_ + ADC_uh_ +K_app_: AUC = 0.923) showed better improvement among three-parameter-based combination models. The paired AUC comparison results derived from the Delong’s test were summarized in **Table S4** and the continuous NRI and IDI were summarized in **Table S5**. Delong’s test result showed that ADC_uh_ at tumor center versus Ki-67 and AQP4 *versus* Ki67 had significant differences (*p* < 0.05) among independent imaging parameters or molecular markers. While for combined models, only ADC_st_+ADC_uh_+K_app_ *versus* K_app_ had a significant difference for AUC comparison (*p* < 0.05). Although the Delong’s test showed no significant AUC difference for some combined models compared with the independent model, significant improvement in discrimination (IDI, *p* < 0.05) and reclassification (NRI, *p* < 0.05) could be obtained by combinations among ADC_st_, ADC_uh_ or K_app_ compared with the independent model, except the comparison between ADC_st_ + ADC_uh_ *versus* ADC_st_ model.

## Discussion

In the current study, DWI parameters metrics derived from low to ultra-high *b* values based on mono-exponential model, IVIM model, DKI model and ultra-high *b*-value model were analyzed from three aspects: (1) their association with expression of histological molecular biomarkers including Ki-67, AQP1, AQP4, and AQP9; (2) if they could be taken as noninvasive surrogated indicators for depicting microenvironment as tumor progression; (3) their preoperative diagnosing ability to differentiate low- and high-grade gliomas.

Our histological results were consistent with previous studies that the AQP1, AQP4 and Ki-67 expression were significantly increased in the high-grade gliomas compared with the low-grade gliomas (31, 32). Our dataset also showed that ADC_st_, ADC_slow_ decreased and ADC_uh_, K_app_ increased significantly in high-grade gliomas compared with low-grade ones.

The alteration of DWI metrics might be explained by molecular origins. The data presented in our study demonstrated that different DWI models fitted within different *b*-value regimes could influence the derived DWI metrics’ correlation level or tendency with histological biomarkers expression. Most of the DWI metrics derived from tumor center had correlations with Ki-67 which indicated tumor cell proliferation-related diffusion restriction effect (33). The ADC_slow_ and ADC_uh_ showed stronger correlations with Ki-67 than other DWI metrics in the current study. It was consistent with previous studies that ADC_slow_ eliminated the effect of microcirculation and could better reflect the water restriction from tumor density and extracellular volume or deposition changes during tumor proliferation (4, 34). It has been also reported that ADC value obtained from high-*b* value (*b* = 3000 sec/mm^2^) had a stronger correlation with Ki-67 index, which was in agreement with the current result for ADC_uh_ (35). ADC_uh_ might give more chance to indirectly reflect the slower diffusion component involved in Ki-67-related proliferation.

The ADC_st_, ADC_slow_ and ADC_uh_ presented correlations with AQP1 and AQP4 in the current study, while AQP9 had no correlation with any DWI metrics. The ADC_st_ and ADC_uh_ presented stronger correlations with AQP1 expression. For AQP4, the ADC_st_ and ADC_slow_ showed stronger correlations.

With the knowledge of slower water transportation speed (36) resulted from the AQPs, previous studies indicated that ADC derived in the high-*b* value range might be one potential marker for AQPs expressions in gliomas (5) or other diseases (25, 37). However, our results showed that the ADC_uh_ was not completely independent from other DWI metrics such as ADC_st_ to independently indicate AQPs expression. And there were already a few studies that reported a negative correlation between ADC_st_ and AQP4 during brain injury or ischemia, which were hypothesized to be associated with decreases in the extracellular space caused by cell swelling (28, 38). The water diffusion condition was originated from the combined contribution of the water exchange effect by AQP overexpression and the proliferation effect by Ki-67. Up-regulations of AQP1 and AQP4 could enhance migration and invasion of glioma cells. In the migration or invasion process, the AQPs might be involved in or play key roles in coordinated cell volume changes (39), reduced tumor cell adhesion with surrounding cells (40), degradation of extracellular matrix (41) and rapid transmembrane water fluxes during lamellipodia formations of glioma cells (14). In addition, AQP1 upregulation is associated with angiogenesis (32) and tumor-associated edema formation (42) and AQP4 redistribution might be functional in the reabsorption of excess cerebral fluid during vasogenic edema (38, 43, 44). These processes might influence the water diffusion in the tissue and transmembrane or microcirculation. In addition, mean kurtosis, as one potential DWI metric measuring the degree of diffusion hindrance or restriction, has been reported to correlate with AQP4 expression in gliomas (21). In the current dataset, the K_app_ showed a larger value in high-grade gliomas which was in accordance with previous studies (45, 46). However, it only showed a significant weak positive correlation with Ki-67 which indirectly reflects proliferation-related diffusion barriers (47). No significant correlation with AQP1 or AQP4 was found for K_app_ in the current research. As parameters derived from models involving high-*b* value, ADC_uh_ and K_app_ had different correlation levels with AQPs which might be resulted from the different *b*-value range for fitting. But the current dataset could not provide sufficient explanations for such deviation. More pathological evidences and a larger sample size should be supplemented to reveal the actual mechanism for AQP-related diffusion effect.

The statistical differences between the DWI metrics derived from tumor center and peritumoral area were analyzed to explore if DWI metrics could be useful to noninvasively describe the glioma microenvironment. In the current study, as gliomas grade increased, the DWI-derived parameters in the tumor center deviated more significantly from those in the peritumoral area. The ADC_st_ at the center of the tumor decreased significantly than that of the peritumoral area in high-grade gliomas which might be a good indicator for the enhanced cellularity in the solid tumor center (48). However, the ADC_st_ might ignore the perfusion effect resulted from tumor vasculature. Therefore, bi-exponential IVIM model was used to distinguish the perfusion and diffusion effects. As expected, the ADC_fast_ increased, ADC_slow_ decreased and K_app_ increased respectively at tumor center compared with those at the peritumoral area in the high-grade gliomas whereas these differences were not significant in the low-grade gliomas. Firstly, the decrease of “true” diffusion coefficient ADC_slow_ and K_app_ in the tumor center might represent the more complicated or heterogenous tumor microstructure, which was in accordance with proliferation-related Ki-67 expression (49). Secondly, as ADC_fast_ (*b* < 200 sec/mm^2^) was able to reflect effective perfusion index (50), the increase of ADC_fast_ at tumor center might be well correlated to perfusion-related angiogenesis of gliomas, which was consistent with the previous study (24). It has been reported that the growth of malignant glioma (WHO grade III and IV) is dependent on new neovascularization which may result in increased permeability, blood flow and transport properties (51, 52). The relative cerebral blood volume increased more frequently in the high-grade gliomas than in the low-grade gliomas (53). Moreover, the AQP1 upregulation might also contribute to the angiogenesis in high grade gliomas (41). The volume fraction of fast pool *f* is mainly affected by the fraction of capillaries and microcirculations. But the *f* parameter at tumor center and peritumor showed an inverse tendency compared with perfusion-related ADC_fast_, in which the *f* became smaller at tumor center as glioma grade increase. Such controversy between *f* and ADC_fast_ existed in a previous report (24). The possible reason for this is that the IVIM model doesn’t involve the influence of echo time to *f*. The T2 value of brain tissues including white or grey matter under 3.0T MRI is greatly deviated from blood’s T2 value. Such deviation might be the reason for the uncertainty of *f* value. Although the current study showed correlations for AQP expression with ADC_uh_, ADC_st_ or ADC_slow_, we still cannot infer that the AQP1 or AQP4 distribution in the tumor center might be denser than peritumor as the immunohistochemical results were only obtained for the solid tumor part. In addition, it has been reported that AQP1 up-regulation is associated with angiogenesis in gliomas and is predominantly located perivascularly and in areas of tumor infiltration whereas distant from the tumor center (32). AQP4 was also reported to have higher expression in both tumor and peritumor than in normal tissues in gliomas, but the degree of peritumoral edema only positively correlates with the expression level of AQP4 in peritumor (9, 22). Therefore, further research is warranted to clarify the spatial distribution of AQPs expressions or tissue arrangement by using pathological, molecular imaging or functional imaging methods and contribute more explanations for DWI metrics.

Finally, the diagnostic performance of the DWI parameters (ADC_st_, ADC_slow,_ ADC_uh_ and K_app_) in differentiating high-grade and low-grade gliomas. We demonstrated that the ADC_st_ had largest AUC 0.833 which was followed by ADC_slow._ While ADC_uh_ had a good sensitivity the same as the ADC_st_. Although there have been reported that conventional ADC_st_ performed well in grading gliomas (47, 54), the added value of other DWI metrics derived from IVIM, ultra-high-*b* value or DKI model is still worthy of study (5, 19, 55). The current research revealed that increased NRI and IDI indices could be obtained by introducing ADC_uh_ and K_app_ into independent ADC_st_ in grading gliomas.

Comprehensive analysis of the relationship between DWI-derived parameters and indices of histopathology has demonstrated the potential of establishing imaging biomarkers reflecting the tumor microenvironment in gliomas. However, further research is required to address several limitations of the present study. Firstly and most importantly, whereas imaging ROIs were placed both at the center of the tumor and in the peritumor, histology assays were not performed separately for these two areas and tumor heterogeneity may have resulted in selection bias. In future studies techniques should be applied so that the imaging and histology refer to the same sampling locations. In addition, by considering the limited sampling area for pathological methods, molecular imaging and functional imaging such as perfusion imaging might be potential methods to help explain the biological significance of DWI metrics. Secondly, the number of patients was relatively small and statistical analysis could not be reliably applied to study different glioma subtypes. As well as studying a larger cohort, additional molecular biomarkers, including proteins and genes, should be included in future studies. Finally, treatment planning must include consideration of regions of glioma infiltration and study of the microenvironment in and beyond the peritumor region is important.

In conclusion, different DWI metrics fitted within different *b*-value ranges (from low to ultra-high *b* values) could act with different efficacy as surrogate indicator for molecular expression or microstructural complexity in gliomas. Further studies which associate pathology or physiology with imaging performance are needed to better explain the biological meanings for these DWI parameters in gliomas.

## Data Availability Statement

The original contributions presented in the study are included in the article/**Supplementary Material**. Further inquiries can be directed to the corresponding author.

## Ethics Statement

The studies involving human participants were reviewed and approved by Institutional Review Board of Henan Provincial People’s Hospital. The patients/participants provided their written informed consent to participate in this study.

## Author Contributions

YB, TL, LC, HG, WW, GZ, SL, HL, and MW designed the overall study, and performed the experiments. YB and MW analyzed imaging data. LW and LK performed histological studies. YB, TL, and MW interpreted the results, and wrote the manuscript. NR revised the language of the manuscript. All authors contributed to the article and approved the submitted version.

## Funding

This work was supported by the National Natural Science Foundation of China (81720108021, 81601466), National Key R&D Program of China (2017YFE0103600), Scientific and Technological Research Project of Henan Province (182102310496), Medical Science and Technology Research Project of Henan Province (2018020403).

## Conflict of Interest

SL and HL are employees of GE Healthcare, China.

The remaining authors declare that the research was conducted in the absence of any commercial or financial relationships that could be construed as a potential conflict of interest.

## Publisher’s Note

All claims expressed in this article are solely those of the authors and do not necessarily represent those of their affiliated organizations, or those of the publisher, the editors and the reviewers. Any product that may be evaluated in this article, or claim that may be made by its manufacturer, is not guaranteed or endorsed by the publisher.
