# Supplementary material for: Study of Diffusion Weighted Imaging Derived Diffusion Parameters as Biomarkers for the Microenvironment in Gliomas
Source: Front Oncol. 2021 Oct 12;11:672265. doi: 10.3389/fonc.2021.672265 (PMC8546342; doi:10.3389/fonc.2021.672265)
Supplement: Supplementary file 1 [file Table_1.docx]

**Supplementary Tables**

**Table S1** The intraclass correlation coefficients (ICC) between the two independent radiologists for measuring different DWI-derived metrics.

|  | **Tumor center** | | **Peritumor** | |
| --- | --- | --- | --- | --- |
| **Variable** | **ICC^a^** | **P-value^b^** | **ICC** | **P-value** |
| ADC_st_ | 0.856 (95%CI: 0.702 - 0.927) | <0.0001 | 0.996 (95%CI: 0.993 - 0.998) | <0.0001 |
| f | 0.853 (95%CI: 0.741 - 0.919) | <0.0001 | 0.999 (95%CI: 0.998 - 0.999) | <0.0001 |
| ADC_fast_ | 0.837 (95%CI: 0.716 - 0.909) | <0.0001 | 1.0 (95%CI: 0.999 - 1) | <0.0001 |
| ADC_slow_ | 0.864 (95%CI: 0.76 - 0.925) | <0.0001 | 0.996 (95%CI: 0.992 - 0.998) | <0.0001 |
| ADC_uh_ | 0.856 (95%CI: 0.746 - 0.92) | <0.0001 | 0.999 (95%CI: 0.998 - 0.999) | <0.0001 |
| K_app_ | 0.987 (95%CI: 0.976 - 0.993) | <0.0001 | 0.972 (95%CI: 0.947 - 0.985) | <0.0001 |

Note:

ADC: apparent diffusion coefficient; ADC_st_: standard apparent diffusion coefficient; ADC_uh_, apparent diffusion coefficient under ultra-high b values; ADC_fast_: pseudo-diffusion coefﬁcient; ADC_slow_: slow diffusion coefficient; *f*: the fraction of fast diffusion component; Kapp: apparent diffusional kurtosis derived from DKI model.

a: intraclass correlation coefficient; b: the statistical significance for ICC analysis, P-value < 0.05 indicated statistical significance.

**Table S2** The correlation coefficient (and P value) derived from the Pearson’s correlation analysis between different DWI-Derived imaging metrics at tumor center.

| **Correlation** | ADC_st_ | f | ADC_slow_ | ADC_uh_ | K_app_ |
| --- | --- | --- | --- | --- | --- |
| ADC_st_ | - | 0.482  (*0.044) | 0.664  (*0.001) | -0.523  (*0.007) | -0.379  (*0.009) |
| f |  | - | 0.724  (*0.0003) | -0.345  (0.256) | -0.345  (*0.036) |
| ADC_slow_ |  |  | - | -0.413  (0.299) | -0.597  (*<0.0001) |
| ADC_uh_ |  |  |  | - | 0.080  (0.772) |
| K_app_ |  |  |  |  | - |

Note:

ADC: apparent diffusion coefficient; ADC_st_: standard apparent diffusion coefficient; ADC_uh_, apparent diffusion coefficient under ultra-high b values; ADC_fast_: pseudo-diffusion coefﬁcient; ADC_slow_: slow diffusion coefficient; *f*: the fraction of fast diffusion component; Kapp: apparent diffusional kurtosis derived from DKI model.

*P-value < 0.05 indicated statistical significance.

**Table S3** The Steiger’s Z test results by comparing different DWI metric’s correlation with the same histological biomarker.

| **Variable** | **Ki-67** | **AQP1** | **AQP4** |
| --- | --- | --- | --- |
| Rst vs. Rslow | 0.692^a^ | 0.367 | 0.986 |
| Rst vs. Ruh | 0.0002 | 0.0002 | 0.0016 |
| Rslow vs. Ruh | <0.0001 | 0.0007 | 0.001 |

Note:

Rst: the correlation between the ADCst with the specific histological biomarker listed in each column.

Rslow: the correlation between the ADCslow with the specific histological biomarker listed in each column.

Ruh: the correlation between the ADCuh with the specific histological biomarker listed in each column.

a: the statistical significance for Steiger’s Z test, P-value < 0.05 indicated statistical significance.

**Table S4** The Delong’s test results for AUC comparisons between paired models in differentiating low- and high-grade gliomas.

| Index | Compared model | Z-statistics | P-value |
| --- | --- | --- | --- |
| 1 | ADCst.vs.ADCslow | 0.224 | 0.823 |
| 2 | ADCst.vs.ADCuh | 0.597 | 0.551 |
| 3 | ADCst.vs.Kapp | 0.910 | 0.363 |
| 4 | ADCst.vs.ADCst+ADCslow | -0.467 | 0.640 |
| 5 | ADCst.vs.ADCst+ADCuh | -1.126 | 0.260 |
| 6 | ADCst.vs.ADCst+Kapp | -0.189 | 0.850 |
| 7 | ADCst.vs.ADCslow+ADCuh | -0.385 | 0.700 |
| 8 | ADCst.vs.ADCslow+Kapp | 0.171 | 0.864 |
| 9 | ADCst.vs.ADCuh+Kapp | -0.511 | 0.609 |
| 10 | ADCst.vs.ADCst+ADCslow+ADCuh | -0.966 | 0.334 |
| 11 | ADCst.vs.ADCst+ADCslow+Kapp | -0.252 | 0.801 |
| 12 | ADCst.vs.ADCst+ADCuh+Kapp | -1.184 | 0.237 |
| 13 | ADCst.vs.ADCslow+ADCuh+Kapp | -0.724 | 0.469 |
| 14 | ADCslow.vs.ADCuh | 0.508 | 0.612 |
| 15 | ADCslow.vs.Kapp | 0.704 | 0.482 |
| 16 | ADCslow.vs.ADCst+ADCslow | -0.725 | 0.468 |
| 17 | ADCslow.vs.ADCst+ADCuh | -1.290 | 0.197 |
| 18 | ADCslow.vs.ADCst+Kapp | -0.320 | 0.749 |
| 19 | ADCslow.vs.ADCslow+ADCuh | -1.016 | 0.310 |
| 20 | ADCslow.vs.ADCslow+Kapp | -0.971 | 0.332 |
| 21 | ADCslow.vs.ADCuh+Kapp | -0.891 | 0.373 |
| 22 | ADCslow.vs.ADCst+ADCslow+ADCuh | -1.877 | 0.060 |
| 23 | ADCslow.vs.ADCst+ADCslow+Kapp | -0.532 | 0.594 |
| 24 | ADCslow.vs.ADCst+ADCuh+Kapp | -1.796 | 0.073 |
| 25 | ADCslow.vs.ADCslow+ADCuh+Kapp | -1.360 | 0.174 |
| 26 | ADCuh.vs.Kapp | 0.030 | 0.976 |
| 27 | ADCuh.vs.ADCst+ADCslow | -0.777 | 0.437 |
| 28 | ADCuh.vs.ADCst+ADCuh | -1.619 | 0.105 |
| 29 | ADCuh.vs.ADCst+Kapp | -0.636 | 0.525 |
| 30 | ADCuh.vs.ADCslow+ADCuh | -1.355 | 0.175 |
| 31 | ADCuh.vs.ADCslow+Kapp | -0.547 | 0.584 |
| 32 | ADCuh.vs.ADCuh+Kapp | -1.709 | 0.087 |
| 33 | ADCuh.vs.ADCst+ADCslow+ADCuh | -1.391 | 0.164 |
| 34 | ADCuh.vs.ADCst+ADCslow+Kapp | -0.684 | 0.494 |
| 35 | ADCuh.vs.ADCst+ADCuh+Kapp | -1.752 | 0.080 |
| 36 | ADCuh.vs.ADCslow+ADCuh+Kapp | -1.724 | 0.085 |
| 37 | Kapp.vs.ADCst+ADCslow | -1.171 | 0.242 |
| 38 | Kapp.vs.ADCst+ADCuh | -1.646 | 0.100 |
| 39 | Kapp.vs.ADCst+Kapp | -1.121 | 0.262 |
| 40 | Kapp.vs.ADCslow+ADCuh | -1.303 | 0.193 |
| 41 | Kapp.vs.ADCslow+Kapp | -0.763 | 0.445 |
| 42 | Kapp.vs.ADCuh+Kapp | -1.553 | 0.120 |
| 43 | Kapp.vs.ADCst+ADCslow+ADCuh | -1.680 | 0.093 |
| 44 | Kapp.vs.ADCst+ADCslow+Kapp | -0.966 | 0.334 |
| 45 | Kapp.vs.ADCst+ADCuh+Kapp | -2.412 | 0.016 |
| 46 | Kapp.vs.ADCslow+ADCuh+Kapp | -1.810 | 0.070 |
| 47 | ADCst+ADCslow.vs.ADCst+ADCuh | -0.886 | 0.376 |
| 48 | ADCst+ADCslow.vs.ADCst+Kapp | 0.422 | 0.673 |
| 49 | ADCst+ADCslow.vs.ADCslow+ADCuh | -0.234 | 0.815 |
| 50 | ADCst+ADCslow.vs.ADCslow+Kapp | 0.636 | 0.525 |
| 51 | ADCst+ADCslow.vs.ADCuh+Kapp | -0.395 | 0.693 |
| 52 | ADCst+ADCslow.vs.ADCst+ADCslow+ADCuh | -1.136 | 0.256 |
| 53 | ADCst+ADCslow.vs.ADCst+ADCslow+Kapp | 0.581 | 0.561 |
| 54 | ADCst+ADCslow.vs.ADCst+ADCuh+Kapp | -1.222 | 0.222 |
| 55 | ADCst+ADCslow.vs.ADCslow+ADCuh+Kapp | -0.667 | 0.505 |
| 56 | ADCst+ADCuh.vs.ADCst+Kapp | 0.982 | 0.326 |
| 57 | ADCst+ADCuh.vs.ADCslow+ADCuh | 0.526 | 0.599 |
| 58 | ADCst+ADCuh.vs.ADCslow+Kapp | 1.241 | 0.214 |
| 59 | ADCst+ADCuh.vs.ADCuh+Kapp | 0.212 | 0.832 |
| 60 | ADCst+ADCuh.vs.ADCst+ADCslow+ADCuh | 0.000 | 1.000 |
| 61 | ADCst+ADCuh.vs.ADCst+ADCslow+Kapp | 0.940 | 0.347 |
| 62 | ADCst+ADCuh.vs.ADCst+ADCuh+Kapp | -0.630 | 0.529 |
| 63 | ADCst+ADCuh.vs.ADCslow+ADCuh+Kapp | -0.048 | 0.962 |
| 64 | ADCst+Kapp.vs.ADCslow+ADCuh | -0.363 | 0.716 |
| 65 | ADCst+Kapp.vs.ADCslow+Kapp | 0.263 | 0.792 |
| 66 | ADCst+Kapp.vs.ADCuh+Kapp | -0.517 | 0.605 |
| 67 | ADCst+Kapp.vs.ADCst+ADCslow+ADCuh | -0.997 | 0.319 |
| 68 | ADCst+Kapp.vs.ADCst+ADCslow+Kapp | -0.135 | 0.893 |
| 69 | ADCst+Kapp.vs.ADCst+ADCuh+Kapp | -1.321 | 0.187 |
| 70 | ADCst+Kapp.vs.ADCslow+ADCuh+Kapp | -0.760 | 0.447 |
| 71 | ADCslow+ADCuh.vs.ADCslow+Kapp | 0.967 | 0.333 |
| 72 | ADCslow+ADCuh.vs.ADCuh+Kapp | -0.398 | 0.690 |
| 73 | ADCslow+ADCuh.vs.ADCst+ADCslow+ADCuh | -0.703 | 0.482 |
| 74 | ADCslow+ADCuh.vs.ADCst+ADCslow+Kapp | 0.317 | 0.751 |
| 75 | ADCslow+ADCuh.vs.ADCst+ADCuh+Kapp | -1.427 | 0.154 |
| 76 | ADCslow+ADCuh.vs.ADCslow+ADCuh+Kapp | -1.427 | 0.154 |
| 77 | ADCslow+Kapp.vs.ADCuh+Kapp | -0.859 | 0.391 |
| 78 | ADCslow+Kapp.vs.ADCst+ADCslow+ADCuh | -1.819 | 0.069 |
| 79 | ADCslow+Kapp.vs.ADCst+ADCslow+Kapp | -0.447 | 0.655 |
| 80 | ADCslow+Kapp.vs.ADCst+ADCuh+Kapp | -1.785 | 0.074 |
| 81 | ADCslow+Kapp.vs.ADCslow+ADCuh+Kapp | -1.334 | 0.182 |
| 82 | ADCuh+Kapp.vs.ADCst+ADCslow+ADCuh | -0.231 | 0.818 |
| 83 | ADCuh+Kapp.vs.ADCst+ADCslow+Kapp | 0.449 | 0.654 |
| 84 | ADCuh+Kapp.vs.ADCst+ADCuh+Kapp | -0.989 | 0.323 |
| 85 | ADCuh+Kapp.vs.ADCslow+ADCuh+Kapp | -0.576 | 0.565 |
| 86 | ADCst+ADCslow+ADCuh.vs.ADCst+ADCslow+Kapp | 1.168 | 0.243 |
| 87 | ADCst+ADCslow+ADCuh.vs.ADCst+ADCuh+Kapp | -0.813 | 0.416 |
| 88 | ADCst+ADCslow+ADCuh.vs.ADCslow+ADCuh+Kapp | -0.061 | 0.951 |
| 89 | ADCst+ADCslow+Kapp.vs.ADCst+ADCuh+Kapp | -1.173 | 0.241 |
| 90 | ADCst+ADCslow+Kapp.vs.ADCslow+ADCuh+Kapp | -0.697 | 0.486 |
| 91 | ADCst+ADCuh+Kapp.vs.ADCslow+ADCuh+Kapp | 0.912 | 0.362 |
| 92 | ADCst.vs.ADCslow | 0.224 | 0.823 |
| 93 | ADCst.vs.ADCuh | 0.597 | 0.551 |
| 94 | ADCst.vs.Kapp_center | 0.910 | 0.363 |
| 95 | ADCst.vs.AQP1 | -0.740 | 0.459 |
| 96 | ADCst.vs.AQP4 | 0.664 | 0.507 |
| 97 | ADCst.vs.AQP9 | 1.401 | 0.161 |
| 98 | ADCst.vs.Ki67 | -1.273 | 0.203 |
| 99 | ADCslow.vs.ADCuh | 0.508 | 0.612 |
| 100 | ADCslow.vs.Kapp_center | 0.704 | 0.482 |
| 101 | ADCslow.vs.AQP1 | -0.909 | 0.363 |
| 102 | ADCslow.vs.AQP4 | 0.490 | 0.624 |
| 103 | ADCslow.vs.AQP9 | 1.433 | 0.152 |
| 104 | ADCslow.vs.Ki67 | -1.578 | 0.115 |
| 105 | ADCuh.vs.Kapp_center | 0.030 | 0.976 |
| 106 | ADCuh.vs.AQP1 | -1.638 | 0.101 |
| 107 | ADCuh.vs.AQP4 | -0.022 | 0.982 |
| 108 | ADCuh.vs.AQP9 | 0.844 | 0.398 |
| 109 | ADCuh.vs.Ki67 | -2.039 | *0.041 |
| 110 | Kapp_center.vs.AQP1 | -1.474 | 0.140 |
| 111 | Kapp_center.vs.AQP4 | -0.054 | 0.957 |
| 112 | Kapp_center.vs.AQP9 | 0.797 | 0.425 |
| 113 | Kapp_center.vs.Ki67 | -1.963 | 0.050 |
| 114 | AQP1.vs.AQP4 | 1.550 | 0.121 |
| 115 | AQP1.vs.AQP9 | 2.475 | *0.013 |
| 116 | AQP1.vs.Ki67 | -0.692 | 0.489 |
| 117 | AQP4.vs.AQP9 | 0.954 | 0.340 |
| 118 | AQP4.vs.Ki67 | -2.040 | *0.041 |
| 119 | AQP9.vs.Ki67 | -3.201 | *0.001 |

*P-value < 0.05 indicated statistical significance.

**Table S5** The continuous net reclassification improvement (NRI) and integrated discrimination improvement (IDI) indices for different models in differentiating high- and low-grade gliomas.

|  | Continuous NRI [95% CI] | P-value | IDI [95% CI] | P-value |
| --- | --- | --- | --- | --- |
| ADC_st__center + ADC_uh__center vs. ADCst | 0.374 [ -0.249 - 0.998] | 0.239 | 0.037 [ -0.054 - 0.127] | 0.425 |
| ADC_st__center + ADC_uh__center vs. ADCuh | 1.138 [ 0.62 - 1.657] | <0.0001 | 0.200 [ 0.042 - 0.358] | 0.013 |
| ADC_uh__center+ Kapp_center vs. ADCuh | 1.195[ 0.710 - 1.679] | 0.000 | 0.200 [ 0.094 - 0.307] | 0.0002 |
| ADC_uh__center+ Kapp_center vs. Kapp | 0.718 [ 0.123 - 1.312] | 0.018 | 0.304 [ 0.144 - 0.464] | 0.0002 |
| ADC_st__center+ ADC_uh__center+ Kapp_center vs. ADCst | 1.061 [ 0.532 - 1.591] | <0.0001 | 0.141 [ 0.035 - 0.246] | 0.009 |
| ADC_st__center+ ADC_uh__center+ Kapp_center vs. ADCuh | 1.215 [ 0.710- 1.721] | 0.000 | 0.304 [ 0.150 - 0.458] | 0.0001 |
| ADC_st__center+ ADC_uh__center+ Kapp_center vs. Kapp | 1.215 [ 0.710 - 1.721] | 0.000 | 0.408 [ 0.257 - 0.559] | 0.000 |

Note:

ADCst: independent ADCst model; ADCuh: independent ADCuh model; Kapp: independent Kapp model; ADC_st_ + ADC_uh_: combination of ADCst and ADCuh model; ADC_st_+ ADC_uh_ +Kapp: combination of ADC_st_, ADC_uh_ and Kapp model. P-value < 0.05 indicated statistical significance.
